# Reconciling the disagreement between observed and simulated temperature responses to deforestation

**DOI:** 10.1038/s41467-019-14017-0

**Published:** 2020-01-10

**Authors:** Liang Chen, Paul A. Dirmeyer

**Affiliations:** 1 Center for Ocean-Land-Atmosphere Studies, George Mason University, Fairfax, VA USA; 2 Climate and Atmospheric Sciences Section, Illinois State Water Survey, Prairie Research Institute, University of Illinois at Urbana-Champaign, Champaign, IL USA

**Keywords:** Atmospheric dynamics, Climate and Earth system modelling, Climate-change impacts

## Abstract

Land use changes have great potential to influence temperature extremes. However, contradictory summer daytime temperature responses to deforestation are reported between observations and climate models. Here we present a pertinent comparison between multiple satellite-based datasets and climate model deforestation experiments. Observationally-based methods rely on a space-for-time assumption, which compares neighboring locations with contrasting land covers as a proxy for land use changes over time without considering possible atmospheric feedbacks. Offline land simulations or subgrid-level analyses agree with observed warming effects only when the space-for-time assumption is replicated. However, deforestation-related cloud and radiation effects manifest in coupled climate simulations and observations at larger scales, which show that a reduction of hot extremes with deforestation – as simulated in a number of CMIP5 models – is possible. Our study provides a design and analysis methodology for land use change studies and highlights the importance of including land-atmosphere coupling, which can alter deforestation-induced temperature changes.

## Abstract (web summary)

Models show a cooler surface temperature response to deforestation than observations which has been attributed to uncertainties in the models. A comparison of satellite observations and model experiments shows that the disagreement is due to the role of atmospheric feedbacks, which are not well captured in the observational space-for-time approach.

## Introduction

The important role of land use/land cover changes (LULCC) has been widely recognized in studies of regional and global climate change. Deforestation, a major type of LULCC due to the harvesting of hardwoods and clearing for agriculture interests, can substantially alter the heat, moisture, carbon, and momentum fluxes between the land surface and atmosphere, and thus influence the atmospheric circulation and climate^1–3^. Compared with the impacts on precipitation, which are more complex and difficult to validate, the relationship between deforestation and temperature has been extensively investigated using both observational datasets and modeling approaches^4^. In particular, recent studies have suggested that historical LULCC (namely deforestation) can significantly influence local high temperature extremes^5–10^.

However, opposite effects of deforestation on summer daytime temperature have been found between most of the state-of-the-art global climate models (GCMs) and observations^11^. Satellite-based observations show that cleared lands are warmer than nearby forest in daily maximum temperature during summer^12–14^. On the contrary, results from CMIP5 (Coupled Model Intercomparison Project Phase 5^15^) and LUCID (land use and climate: identification of robust impacts^16,17^) tend toward a summer daytime cooling over the middle latitudes as a result of documented historical deforestation^6,11^. The disagreement of model results with observations has been claimed to be associated with parameterization issues in current land surface models, such as the changes in partitioning of available energy between latent and sensible heat fluxes due to LULCC^6,8,11^.

Despite existing uncertainties in land surface models^18,19^, the limitations of current comparisons between the observations and model simulations should not be overlooked. Unlike climate models, which incorporate time-evolving or timeslice LULCC scenarios in sensitivity studies with multiple simulations to examine the temperature response to deforestation, observational data are difficult to parse for deforestation signals that are mixed among other climatic factors. Therefore, most of the observation-based studies focus on the contrast between nearby locations that have different land cover (e.g., forest vs. open land^12–14,18,20,21^) or that have undergone different land cover change scenarios (e.g., deforestation vs. afforestation^22,23^). These observational studies using data from flux towers or satellite retrievals contain an assumption that the neighboring land units share the same atmospheric background state and thus differences in surface temperature can be attributed solely to LULCC. This space-for-time analogy works for detecting the local impacts of deforestation, but two issues emerge when comparing the observed local impacts with model simulations. First, the climate model-based sensitivity studies, where separate integrations with and without LULCC are compared, necessarily reflect atmospheric feedbacks (e.g., changes in clouds or precipitation due to LULCC) that are fundamentally different between scenarios, an arrangement that conflicts with the assumptions of local detection of LULCC signals. Background states are not preserved. Second, the climate models have coarse spatial resolutions, while the satellite- or tower-based observational methods are usually applied at much finer scales.

In this study, we test a similar space-for-time approach on different satellite-based products (Supplementary Table 1) to explore the possible relationship between deforestation and boreal summer (June–August) land surface temperature (LST) at different scales (see “Methods”). A set of deforestation simulations is conducted using the Community Earth System Model (CESM^24^) with and without considering atmospheric feedbacks (see “Methods”, Supplementary Table 2) by including a variety of coupled and uncoupled experiments at a low spatial resolution to explore possible approaches to satisfy the space-for-time assumption, and an additional coupled experiment at a high spatial resolution to explore the scale dependency of temperature responses. We find that the impacts of deforestation on surface temperature are scale-dependent. At the local scale without considering atmospheric feedbacks, as the space-for-time assumption is satisfied, the observations and climate model results agree on summer daytime warming caused by deforestation. However, at larger scales, deforestation-related atmospheric feedbacks manifest in coupled climate simulations and observations, which show that deforestation may reduce surface temperature through cloud and radiation effects.

## Results

### Observed sensitivity of LST to deforestation

**Fig. 1:**
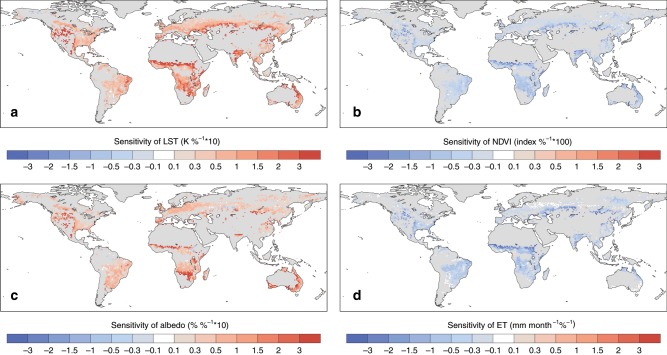
Observed sensitivity to deforestation. **a** summer daytime land surface temperature (LST), **b** normalized difference vegetation index (NDVI), **c** albedo, and **d** evapotranspiration. The sensitivity is calculated based on the 0.05° Moderate Resolution Imaging Spectroradiometer (MODIS) satellite products within a 1° analysis window. The sensitivity is shown only where it is significant at a false discovery rate (FDR) adjusted *p* values of 0.05 and the range of deforestation (maximum minus minimum deforestation within the window) is greater than 10%. The figure was created using the NCAR Command Language (https://www.ncl.ucar.edu).

Figure 1a shows the sensitivity of boreal summer daytime LST to deforestation based on Moderate Resolution Imaging Spectroradiometer (MODIS) observations using the space-for-time approach. Even though disagreement between observations and models also occurs for minimum temperature and temperature in other seasons^25^, most of the inconsistencies that have been discussed regard boreal summer daily maximum temperature or hot extremes, which have drawn increasing attention. Also, because land–atmosphere interactions are particularly strong during the warm season^26,27^, we focus on the summer daytime (or maximum) temperature in this study. Consistent with previous studies^12–14,22^, deforestation tends to increase the local LST globally during summer. Spatially, stronger LST sensitivity is found over arid or semi-arid regions, such as the western USA, India, central Asia, African Savanna, and parts of Australia. Results from the other two high-resolution LST products also suggest local warming effects by deforestation with similar spatial patterns (Supplementary Fig. 1).

Figure 1b–d shows the relationship between deforestation and other land surface quantities. Generally, deforestation corresponds to lower vegetation index (less dense vegetation), higher albedo, and less evapotranspiration. The strength of the sensitivities shows a very similar spatial pattern to the LST response. Because deforestation also leads to changes in surface roughness (e.g., about 1 m decrease for the temperate and boreal deforestation and 2.5 m decrease for the tropical deforestation), affecting sensible heat flux efficiency, it is difficult to conclude based only on current satellite data whether the local daytime warming is associated only with the evaporative feedback.

### Simulated LST responses to deforestation

**Fig. 2:**
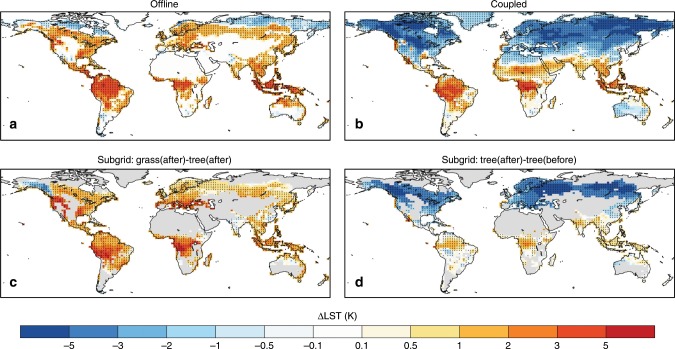
Simulated impacts of deforestation on summer daily maximum land surface temperature. The land surface temperature (LST) difference is calculated based on the Community Earth System Model (CESM) **a** low-res offline, **b** low-res coupled, and (**c**, **d**) subgrid deforestation experiments. In **c** the local impacts are defined as the LST difference between the grass and tree tiles in the postdeforestation (after) simulation; in **d** the nonlocal impacts defined as the LST difference for the tree tiles between the postdeforestation (after) and predeforestation (before) simulations. Stippling indicates the temperature difference is significant at a false discovery rate (FDR) adjusted *p* values of 0.05.

Figure 2 shows the LST changes after deforestation based on climate model simulations (offline, coupled, and subgrid experiments). The offline experiment is performed using only the land surface model driven by observed atmospheric forcings; the coupled experiment is performed using the coupled land-atmosphere model; the subgrid experiment also uses the coupled model but the analysis is performed at subgrid level with the focus on the vegetation tiles within each grid cell (see “Methods”). Even though there are identical deforestation scenarios prescribed in the offline and coupled experiments, we see very different LST responses, particularly over the middle and high latitudes. In the offline experiment, deforestation increases LST globally (except some areas in high latitudes due to snow-albedo effects). According to previous studies, the warming effects of tropical deforestation are mainly associated with decreased evapotranspiration^28^. Over the temperate and boreal forests, the warming effects are mainly attributed to the changes in surface roughness, because the aerodynamically smoother surface of the open lands hinders the turbulent transfer of heat to the atmosphere^28^. However, in the coupled simulations, significant surface cooling is found over most areas of the middle and high latitudes (Fig. 2b), which agrees with the cooling effects from historical LULCC in the CMIP5 simulations^7,11^. The offline simulation effectively shares the same assumption as the space-for-time approach applied to the observations, because the atmospheric forcings to the offline land surface model are identical with and without deforestation. Therefore, the different LST responses in the offline and coupled experiments indicate that the atmospheric feedbacks can overwhelm the local warming effects when deforestation is widespread, obviating the space-for-time assumption.

Another way to estimate the LST responses to deforestation is by comparing simulated LST across different land-use tiles within a model grid cell^29,30^, which allows separation of the local effects and atmospheric feedbacks in the coupled simulations. Within a grid cell, the difference in LST between tree and grass tiles in the deforestation simulation can be considered as the result of local land use changes (Fig. 2c), as they share the same atmospheric column but are different land units, analogous to the offline experiments. It should be noted that the different land cover tiles still share the same soil column, which can influence the calculated temperature at each tile. Thus, a previous study^19^ with a more realistic soil column parameterization suggests an enhanced contrast between forest and open land. Nevertheless, the subgrid soil column issue is not likely to affect the deforestation-induced local warming identified in this study. Meanwhile, the difference in LST for the same tree tiles between the control and deforestation simulations can be considered as the result of the atmospheric feedbacks (Fig. 2d), because they are the same land units receiving altered atmospheric forcings. Overall, the atmospheric feedbacks indicate a slight warming in the tropics, but a strong cooling over the middle and higher latitudes, which exceeds the local warming due to deforestation.

From this perspective, the CESM (or Community Land Model, CLM) simulations (subgrid or offline comparisons) show consistency with the observations when they are considered on par with the land use change impacts measured in observations. This suggests that the disagreement with observations regarding summer daytime cooling over the middle latitudes found in climate models may be due to the role of atmospheric feedbacks in the coupled simulations, rather than uncertainties or errors in the land surface models. The contrast between local and nonlocal effects has been documented in other climate models^30–32^.

### A fair comparison for the coupled experiments

**Fig. 3:**
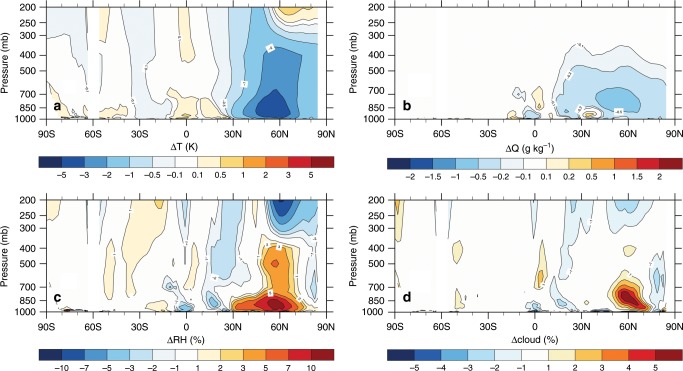
Simulated impacts of deforestation on the atmosphere based on the coupled deforestation experiments. Changes in zonal-mean (land only) **a** temperature (T), **b** specific humidity (Q), **c** relative humidity (RH), and **d** cloud cover after deforestation.

**Fig. 4:**
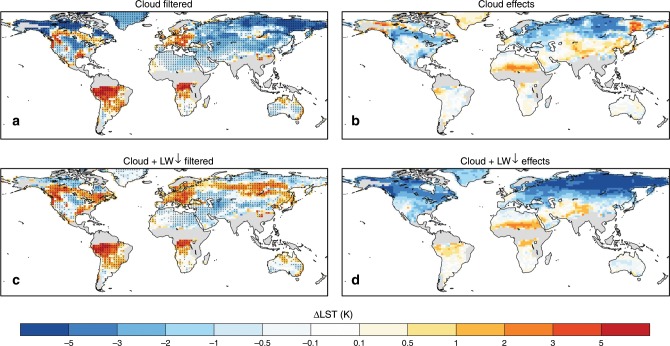
Simulated impacts of deforestation on summer daily maximum land surface temperature based on the coupled deforestation experiments. **a** Change in land surface temperature (LST) without cloud effects and **c** without additional downward longwave (LW↓) effects. Two filters (see “Methods”) were applied to the coupled deforestation experiments to limit the effects of **b** cloud and **d** downward longwave (LW↓) on land surface temperature. Stippling on **a** and **c** indicates the temperature difference is significant at a false discovery rate (FDR) adjusted *p* values of 0.05.

To investigate the full impacts of LULCC, one must not ignore the atmospheric feedbacks. In coupled deforestation experiments, we see significant responses in the lower atmosphere northwards of 30 °N, such as decreased temperature and specific humidity, and increased relative humidity and cloud fraction (Fig. 3). Here, we present filtered LST changes by systematically accounting for, and removing, the influence of changes in the atmospheric background states in the coupled experiments (Fig. 4), so that they become comparable to observational space-for-time LULCC assessments.

First, optical satellite observations of the surface are usually restricted to nonovercast conditions. Therefore, we apply the first filter keeping only records of daily maximum LST under clear-sky conditions (Fig. 4a), so that the impacts of clouds on downward shortwave (SW) and longwave (LW) radiation, as well as immediate effects of precipitation, can be accounted for. Under clear-sky conditions, increased LST is found in limited areas over North America and Europe, but there remains overall cooling over middle and high latitudes.

Meanwhile, due to the cooler atmosphere after deforestation (Fig. 3), we add a second filter (see “Methods”) to remove the additional influence caused by differences in downward LW radiation (Fig. 4c). Under similar atmospheric conditions, coupled simulations show very similar surface warming from deforestation compared with the offline (Fig. 2a) or subgrid (Fig. 2c) simulations, further demonstrating that simulated LST responses to deforestation in climate models can agree with observations as long as the underlying assumptions are consistent.

### Scrutinizing the space-for-time assumption

**Fig. 5:**
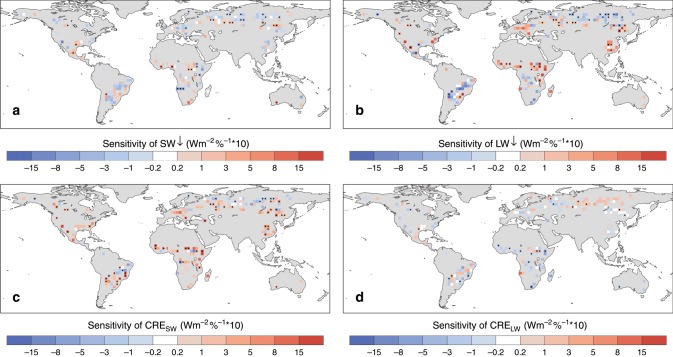
Observed sensitivity of radiation and cloud radiative effects to deforestation. **a** Clear-sky downward shortwave (SW↓), **b** clear-sky downward longwave (LW↓) radiation, and the cloud radiative effects on net **c** shortwave and **d** longwave radiation. The sensitivity is calculated based on the Clouds and the Earth’s Radiant Energy System (CERES) data within a 3° × 3° analysis window. The black dot signs indicate the sensitivity is significant at a false discovery rate (FDR) adjusted *p* values of 0.05.

For the space-for-time comparisons based on either paired in situ measurements or high-resolution satellite observations, it is assumed that the paired sites or pixels within the analysis windows have the same atmospheric background states. However, there is a lack of in situ observations to verify how different the atmospheric conditions can be between forest and open lands at the local scale. Using measurements of surface radiation (with 1° spatial resolution) from the Clouds and the Earth’s Radiant Energy System (CERES) project^33^, we estimate the possible sensitivity (see “Methods”) of downward radiation and clouds to deforestation at a broader scale (Fig. 5), which is more comparable to the scale of climate models. Under clear-sky conditions, there tends to be less incoming SW radiation over a majority of the deforested areas in the middle and high latitudes, but mixed signals in the tropics (Fig. 5a). Deforestation corresponds to higher downward LW radiation over the central US, Europe, eastern China, and southern Sahara, but significantly less LW radiation over Siberia, central/southern Africa, and Brazil (Fig. 5b).

Figure 5c, d shows the cloud radiative effect (CRE) on net SW and LW radiation, respectively. Generally, CRE tends to increase the SW radiation and decrease the LW radiation in most of the regions in the middle and low latitudes, indicating a possible decrease in cloud cover over the deforested areas. However, decreased SW and increased LW are found over Siberia and parts of South America, implying that deforestation may increase cloud cover in these regions. This deforestation-cloud relationship is further confirmed when applying the analysis on the MODIS monthly cloud fraction product (MOD08_M3^34^, Supplementary Fig. 2). As we see significant difference in the incoming radiation and cloud cover associated with deforestation, the space-for-time assumption may not be applicable at broader scales.

### Detecting the LST responses at different scales

**Fig. 6:**
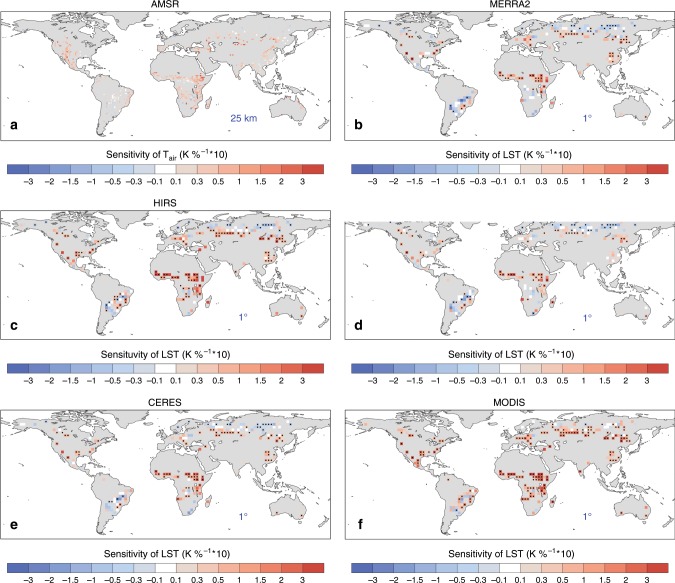
Observed sensitivity of surface temperature to deforestation using different satellite and reanalysis products at different scales. **a** Daily maximum 2-m air temperature derived from the Advanced Microwave Scanning Radiometer-Earth Observing System and Advanced Microwave Scanning Radiometer 2 (AMSR-E and AMSR2), **b** land surface temperature (LST) from Modern-Era Retrospective analysis for Research and Applications, Version 2 (MERRA-2) reanalysis, **c** daily maximum LST from the Princeton High-Resolution Infrared Radiation Sounder (HIRS) products, **d** skin temperature from ERA-Interim, **e** LST derived from the Clouds and the Earth’s Radiant Energy System (CERES) upward longwave radiation, and **f** daytime LST aggregated from the Moderate Resolution Imaging Spectroradiometer (MODIS) LST product. Blue text shows the spatial resolutions of the temperature datasets. The AMSR surface temperature was analyzed within 125 × 125 km windows, and the other temperature datasets were analyzed within 3° × 3° windows. For panel **a**, the sensitivity is shown only where it is significant at a false discovery rate (FDR) adjusted *p* values of 0.05. The black dot signs in **b**–**f** indicate the sensitivity is significant at an FDR adjusted *p* values of 0.05.

When applying the space-for-time method to coarse-resolution (0.5–1°) satellite and reanalysis datasets (Fig. 6), LST has a different sensitivity to deforestation compared with the overall warming seen from the high-resolution satellite observations (Fig. 1 and Supplementary Fig. 1). Although the coarse-resolution observations may be less representative of the land cover information, similar space-for-time methods have been applied to climate model output with coarser spatial resolutions and larger analysis windows in previous studies to identify the deforestation-induced signals^9,11,35^. Generally, deforestation-related warming is found in most of the areas in the middle and low latitudes of the Northern Hemisphere; but cooling effects are observed in the boreal forest, parts of Brazil and southern Africa.

Even though uncertainty exists among the different temperature datasets and temperature variables from reanalysis products can be affected by model artifacts, we see similarities in the spatial patterns of temperature sensitivities in Fig. 6 and atmosphere sensitivities Fig. 5. Over the regions with deforestation-related decreased downward LW radiation and increased cloud cover such as parts of South America and Siberia, cooling effects become evident from the coarse-resolution observations. In other regions with deforestation-related increased downward radiation and decreased cloud cover, warming effects remain in the coarse-resolution observations. It should be noted that the local warming from the high-resolution satellite observations is under clear-sky conditions, but regional signals from the coarse-resolution datasets (AMSR, MERRA-2, Princeton HIRS, ERA-Interim, and CERES) usually include cloud effects. Due to the competition between the cooling effects through reduced downward shortwave radiation and the warming effects through increased downward LW radiation, we found that the cloud effects on LST sensitivities are relatively small based on the clear-sky and all-sky CERES products (Supplementary Fig. 3). Therefore, the difference in detected LST responses at different scales (in Figs. 1 and 6) suggest that the emerging atmospheric feedbacks at a broader scale, especially the downward LW radiation, are able to modulate the local impacts of deforestation. This can also be confirmed by the comparison between MODIS-based LST at different scales (Figs. 1a and  6f), which shows different clear-sky LST sensitivities to deforestation in parts of South America and Siberia. Furthermore, for the observations at an intermediate resolution (e.g., AMSR in Fig. 6a), the cooling effects are less evident than the low-resolution observations. Although the difference may be related to the different temperature variable measured in AMSR compared with other datasets, this also suggests that the atmospheric feedback is scale-dependent and it is not as strong as that identified in the low-resolution datasets.

There are several possible reasons to explain the deforestation-induced atmospheric cooling. For instance, Siberian deforestation leads to a local warming (Fig. 1a), but the increase in surface albedo (Fig. 1c) can modify the temperature and humidity throughout the troposphere (Fig. 3), cooling the land surface through atmospheric feedback. This deforestation-related decrease in radiative forcing in high latitudes is also reported in previous studies either using observations^36^ or climate models^1,37–39^. In South America and southern Africa, significantly enhanced shallow cloud cover has been found over the deforested (or savanna) areas during the dry season^40–43^, which can lead to land surface cooling. In addition, tropical deforestation-related cooling is apparent over the regions with frequent fires^44,45^, which generate abundant aerosols, affecting the atmospheric radiative balance and potentially cloud formation and rainfall^3,46^. Although the mechanism of atmospheric feedback needs further detailed investigation in future studies, the observed dominance of atmospheric feedback is replicated in the CESM experiments, in which the atmospheric feedback in the coupled simulations overwhelms the local warming in the offline simulations. Furthermore, different LST responses to deforestation are also found in the climate model simulations at different resolutions (Supplementary Fig. 4). The atmospheric effects become more pronounced as the grid size increases—registering stronger cooling from deforestation over the middle and high latitudes and stronger warming over the tropics.

## Discussion

We acknowledge that the sign of atmospheric feedbacks can be different between observations and model simulations, particularly over the tropics. It should be noted that deforestation can also affect temperature through mesoscale circulation^40–43^ and fire/aerosol feedback^44,45^, but these processes are not represented either in the offline or coupled GCM experiments. The cooling effects over Brazil and central/southern Africa identified in the observations can be associated with changes in mesoscale circulations due to the contrasting surface conditions^3^ that cannot be explicitly represented in a low-resolution climate model. However, both observations and model simulations exhibit a similar scale dependency of LST response, suggesting that the local warming from deforestation can be weakened or masked by atmospheric feedbacks when the scales are broader. Moreover, the complete deforestation scenario used in this study amplifies the atmospheric responses, which can depend on the scale of the deforestation^47^. Therefore, the observed and simulated atmospheric effects can be different, such as the simulated strong atmospheric cooling in the middle and high latitudes, and the different cloud cover changes in the observations and coupled simulations.

Furthermore, this is not a single-model issue. Contrasting local and nonlocal LST effects have also been documented in CMIP5 participating models other than CESM (e.g., GFDL^30^ and MPI-ESM^31,32^). Therefore, the methodology for a fair comparison in this study can be applied to model evaluation and intercomparison in CMIP6 experiments like the Land Use Model Intercomparison Project^48^. Despite the uncertainties in current land surface models^19^, the revealed agreement in the sign of temperature changes in our study highlights the need for such fair comparisons in future model evaluations and LU-impact investigations. The disagreement in land-use induced summer daytime temperature responses between current climate models and observations may reflect the uncertainties in land surface model^6^ and the choice of temperature variables^49,50^. However, attention should also be paid to atmospheric model responses and the coupling between land and atmosphere, because atmospheric feedback to LULCC can dominate the deforestation-induced LST changes.

Lastly, the authors acknowledge the limitations of this study, especially the uncertainties in the observational datasets. For instance, the atmospheric feedback estimate mainly relies on the CERES dataset, which has a relatively coarse spatial resolution and uncertainties in cloud properties and surface irradiances. Meanwhile, the reanalysis datasets may not fully represent the land surface conditions within their grid cells, because most standard observations including those assimilated by the reanalysis are collected over open land and thus not representative of forest conditions. Therefore, more satellite-based observations of the atmosphere, especially the boundary layer, at a higher spatial resolution are needed in future studies to further understand the land-atmosphere feedback in the context of land use or deforestation.

In summary, this study presents a fairer comparison of the LST responses to deforestation between the observations and model simulations. The subgrid comparison and the filters in the coupled simulations can separate the local and nonlocal impacts of deforestation. At the local scale, as the space-for-time assumption is then satisfied, the climate model results agree better with the observed summer daytime warming caused by deforestation. The commonly mentioned disagreement (e.g., refs. ^8,9,11^) in previous studies is mainly associated with neglected atmospheric feedbacks, which cannot be directly compared with observations in part because of differences in scale. These findings reveal that the cooling effect of deforestation (or historical LULCC) on hot extremes is still possible, as shown in a number of the CMIP5 models.

## Methods

### Observed temperature response to deforestation

The summer daytime LST climatology is calculated based on the MODIS/Aqua MYD11C2 V006 LST 8-day composite product at 0.05° × 0.05° resolution over the period 2002–2017. Pixels with water bodies and snow cover (if greater than 0.1% of the grid cell) are excluded in the analysis. Percentage of water bodies is calculated based on the MODIS MCD12C1 land cover type yearly product^51^ with the IGBP land cover classification during the period 2001–2012; and summertime snow cover is calculated based on the MODIS/Terra MOD10CM snow cover monthly product^52^ during the period 2002–2017. Considering the possible effects of complex topography, we have tried correcting the LST based on the average climate lapse rates derived from ERA-Interim reanalysis, but the correction had little influence on the results, thus it is not included in the analysis.

The forest cover is derived from the tree canopy cover for year 2000 from the Global Forest Change 2000–2016 dataset^53^, which is aggregated from 1″ (about 30 m) to the MODIS resolution. We subtract the forest cover (percentage area of forest in a pixel in the range 0–100) from 100 to obtain the percentage of area with deforestation or tree loss. Within a 1° × 1° analysis window, we assume all the pixels share the same atmospheric background states. If the range of deforestation (maximum minus minimum deforestation within the window) is greater than 10% (to ensure contrasting land cover conditions), we calculate the slope of the linear regression of LST on deforestation, which can be considered as the local sensitivity of LST to tree loss. The same method is applied to the other two high-resolution satellite LST datasets (ATCDR and Merged GEO), as well as MODIS NDVI, albedo and evapotranspiration products. For other temperature products with a coarser resolution, we regrid the land cover and snow cover data to the same resolution as the temperature products, and use certain snow and water filtering criteria and analysis window sizes to estimate the surface temperature sensitivity at different scales (Supplementary Table 1). Similarly, for coarse-resolution datasets (AMSR, MERRA-2, Princeton HIRS, ERA-Interim, and CERES), the sensitivity of temperature and other environmental variables to deforestation is calculated based on the slope of their linear regression on deforestation within a 3° × 3° analysis window. Statistical significance of the linear regression between LST (or other environmental variables) and deforestation is determined by the Student’s *t* test. Conducting significance tests separately at each grid point can increase the probability of false rejection of the null hypothesis^54^. We therefore control the false discovery rate with the approach described by ref. ^55^, using a global *p* value of 5 %.

The largest window size is 3° × 3° in the sensitivity analysis based on the linear regression of environmental variables on deforestation. Previous studies^11,35^, which also use the space-for-time assumption, have even coarser spatial resolution (e.g., minimum window size by 5 × 5 grid cells of the GCMs in ref. ^11^; minimum window size by 10° × 10° and maximum size by 15° × 20° in ref. ^35^). Although the broader scale (e.g., within a 3° × 3° analysis window) may be too coarse to capture the local effects of deforestation, we are able to detect the sensitivities of atmospheric background to tree cover (e.g., Fig. 5). Moreover, it should be noted that the low-resolution observations may be less effective at representing the land surface information; higher resolution radiation flux observations are needed to confirm the relationship between the local effects of deforestation and atmospheric feedbacks.

### Model and experiments

A set of experiments are performed with the CESM version 1.2.2 to investigate the impacts of deforestation on surface temperature (Supplementary Table 2). The component set F_2000, which includes Community Atmosphere Model version 4 (CAM4)^56^ and the Community Land Model 4.0 (CLM4)^57^ with prescribed satellite phenology, has been applied at two different horizontal resolutions (high resolution: f05_g16, 0.47° × 0.63° and low resolution: f19_g16, 1.9° × 2.5°). We have also used CAM5 as the atmospheric model, which produces very similar results (not shown). Previous studies have suggested the possible impacts of deforestation on temperature through oceanic feedback^38,58^, but the focus of this study is on the local impacts at the land surface and nonlocal impacts from the atmospheric feedback. Therefore, for computational efficiency especially for the high-resolution simulations, the coupled experiments are climatology simulations with cyclic circa-year-2000 forcing with identical prescribed sea surface temperature (SST) and sea ice cover climatologies^59^ with a fixed CO_2_ concentration of 367.0 ppm. For the local impacts of deforestation, offline deforestation experiments have also been conducted using CLM4 with CRUNCEP atmospheric forcings from 1951–2010^60^.

In CLM, spatial land surface heterogeneity is represented as a nested subgrid hierarchy, in which grid cells are composed of multiple land units, snow/soil columns, and plant functional types (PFTs). First, pre-deforestation land cover conditions are generated at 0.47° × 0.63° resolution based on the pre-industrial (1850) land cover dataset provided in CESM^61^. We keep only the dominant PFT within a single grid cell, so that there is an explicit land cover assigned for each grid cell. Second, we replace all the tree PFTs with grass PFTs for the postdeforestation land cover condition. The types of grass are determined by latitude: C4 grass between 30 °S and 30 °N, C3 grass between 30° and 60°, and arctic C3 grass beyond 60°. Lastly, we aggregated the high-resolution land surface data from 0.47° × 0.63° to 1.9° × 2.5°, so that the low-resolution experiments have the identical subgrid vegetation changes as the high-resolution experiments. The temperature difference between the pre-deforestation and postdeforestation simulations can be considered as the impacts of globally complete deforestation.

For the subgrid comparison, we have conducted another deforestation experiment with PFT-level output for each grid cell at the 1.9° × 2.5° resolution. The land surface data for the predeforestation condition are based on the pre-industrial (1850) land cover dataset. For the postdeforestation condition, the total area of the tree PFTs was reduced to a small nonzero area (0.01%) of the grid cell, and the remaining area is replaced by grass PFTs. For a grid cell with multiple tree PFTs, their areas are reduced proportionally based on their predeforestation conditions. Because the land model will not perform calculations for the PFTs that are completely removed from a grid cell, this modification ensures that the simulation can be consistent with the complete deforestation experiment above, yet calculations can be performed on the tree PFTs for our subgrid comparison. We compare the temperature changes from the subgrid experiments with the complete deforestation experiment at the grid level. Subtle differences are found between these two experiments. Previous studies have reported a soil column sharing issue in CLM^19,29^, which can have substantial influence on the ground heat flux and temperature at the subgrid level. However, the temperature contrast between the tree and grass PFTs is not affected in terms of the sign of temperature changes (according to ref. ^29^). With the output for individual PFTs in each grid cell, we can identify the local impacts of deforestation based on the difference between the tree and grass PFTs in the postdeforestation simulation. It is called local because the tree and grass PFTs always receive the same atmospheric forcing within a grid cell. Meanwhile, the nonlocal impacts of deforestation, which are induced by the changes in the atmospheric background, can be identified based on the temperature difference for the same tree tiles between the postdeforestation and predeforestation simulations.

For all the experiments, the land initial conditions have been generated through 40-year offline CLM simulations, and then used for the coupled and offline experiments. We run all experiment simulations for 60 years, with 3-hourly output. Because the satellite-based temperatures are land surface temperatures, and inconsistent responses to deforestation are found between land surface temperature and air temperature^49,50^, we use radiative surface temperature (a.k.a. skin temperature) from the model to maintain consistency. We identify the summer daily maximum LST, and the corresponding cloud cover and surface fluxes when the LST reaches the daily maximum.

### Filters in the coupled experiments for a fair comparison

Due to the changes in atmospheric background states as a result of feedbacks from LULCC, the results from the coupled experiments cannot be directly compared with the observations. To ensure the space-for-time assumption can be satisfied, we applied two filters to limit the impacts of atmospheric feedback on LST. In each simulation, there are 5520 summertime records (92 days × 60 years) at each grid cell. First, daily records with cloud cover greater than 0.01% were excluded to ensure clear-sky conditions. This filter removes the impacts of changes due to cloud cover and active precipitation.

Because deforestation leads to a significant cooling for the whole atmosphere in middle and high latitudes that can reduce downward LW radiation even under the clear-sky conditions, a second filter is applied to minimize the impacts of downward LW radiation on LST. At each grid cell, we combine all the records of clear-sky downward LW radiation from both the pre-deforestation and postdeforestation simulations and calculated the lower tertile and upper tertile of the combined records. Then, only records with downward LW radiation that falls in the joint middle tertile are retained for the analysis. This filter helps ensure the two simulations have comparable downward LW radiation.

## Supplementary information

Supplementary Information

## Data Availability

The observational and reanalysis data used in this study are publicly available. The MODIS products, used for Figs. 1, and 6f, were retrieved from the online Data Pool, courtesy of the NASA Land Processes Distributed Active Archive Center (LP DAAC), USGS/Earth Resources Observation and Science (EROS) Center, Sioux Falls, South Dakota, https://lpdaac.usgs.gov. The ATCDR and Merged GEO LST products, used in Supplementary Fig. 1, were obtained from the GlobTemperature Data Portal (http://data.globtemperature.info/). Daily Global Land Parameters Derived from AMSR-E and AMSR2, Version 2, underlying Fig. 6a, are obtained from NASA National Snow and Ice Data Center Distributed Active Archive Center. MERRA-2 data for Fig. 6b are available at MDISC managed by the NASA Goddard Earth Sciences (GES) Data and Information Services Center (DISC). The LST dataset based on the High-Resolution Infrared Radiation Sounder (HIRS) for Fig. 6c was obtained from http://hydrology.princeton.edu/data.lst.php. EAR-Interim reanalysis used for Fig. 6d were obtained at European Centre for Medium-Range Weather Forecasts. The CERES datasets for Figs. 5, 6e, and Supplementary Fig. 3 were obtained from http://ceres.larc.nasa.gov/order_data.php. MODIS monthly cloud fraction product (MOD08_M3) underlying Supplementary Fig. 2 was obtained from The Level-1 and Atmosphere Archive & Distribution System (LAADS) Distributed Active Archive Center (DAAC), https://ladsweb.modaps.eosdis.nasa.gov/. The Community Earth System Model is freely available at http://www.cesm.ucar.edu/models/cesm1.2/. All CESM results used for Figs. 2, 3, 4, and Supplementary Fig. 4 are available from the authors on reasonable request.

## Code availability

All the climate model output and NCL codes used for the numerical procedures are available from the corresponding author upon reasonable request.
