# Supplementary material for: Reconciling the disagreement between observed and simulated temperature responses to deforestation
Source: Nat Commun. 2020 Jan 10;11:202. doi: 10.1038/s41467-019-14017-0 (PMC6954270; doi:10.1038/s41467-019-14017-0)
Supplement: Supplementary file 1 — Supplementary Information [file 41467_2019_14017_MOESM1_ESM.pdf]

Supplementary information for:

Reconciling the Disagreement between Observed and Simulated Temperature Responses to Deforestation

By Liang Chen and Paul A. Dirmeyer

Supplementary Table 1: Surface temperature data used for calculating the sensitivity to deforestation

Name	Source	Period	Resolution	Analysis window size	Criteria to mask water	Criteria to mask snow	Minimum sample size
MODIS	MODIS/Aqua MYD11C2 V006 LST 8-day composite product[1]	2002-2017	0.05°×0.05°	1° × 1°	< 0.1%	< 0.1%	200
MODIS	MODIS MOD13C1 V006 vegetation indices 16-day product[2]	2002-2017	0.05°×0.05°	1° × 1°	< 0.1%	< 0.1%	200
MODIS	MODIS MCD43C3 V006 albedo 16-day composite product[3]	2002-2017	0.05°×0.05°	1° × 1°	< 0.1%	< 0.1%	200
MODIS	MODIS global evapotranspiration project (MOD16) monthly product[4]	2002-2017	0.05°×0.05°	1° × 1°	< 0.1%	< 0.1%	200
ATCDR	the ATSR Climate Data Record (CDR) LST product[a]	2000-2011	0.05°×0.05°	1° × 1°	< 0.1%	< 0.1%	200
Merged GEO	The geostationary satellites LST merged product[b]	2011-2013	0.05°×0.05°	1° × 1°	< 0.1%	< 0.1%	200
AMSR[5]	Daily maximum 2m air temperature from AMSR-E and AMSR2	2002-2017	25 km × 25 km	125 km × 125 km	< 5%	< 0.5%	20
MERRA-2[6]	Monthly surface skin temperature from the MERRA-2 reanalysis	2002-2017	0.5°×0.5° (regridded to 1°×1°)	3° × 3°	< 5%	< 1%	9
Princeton HIRS[7]	Daily maximum LST from the HIRS retrievals combined with NCEP/CFSR	2000-2009	0.5°×0.5° (regridded to 1°×1°)	3° × 3°	< 5%	< 1%	9
ERA-Interim[8]	Monthly surface skin temperature from the ERA-Interim reanalysis	2002-2017	0.75°×0.75° (regridded to 1°×1°)	3° × 3°	< 5%	< 1%	9
CERES[9]	Calculated LST based on monthly all-sky upward longwave radiation in CERES	2005-2015	1°×1°	3° × 3°	< 5%	< 1%	9

a. The Advanced Along Track Scanning Radiometer (AATSR) LST products are made available through the GlobTemperature data portal with the support of the European Space Agency (ESA) and the UK National Centre for Earth Observation (NCEO).

b. The LST data derived from SEVIRI/Meteosat is entirely based on the LST product generated within the EUMETSAT Satellite Applications Facility on Land Surface Analysis (LSA SAF product LSA-001). The LST data derived from GOES and MTSAT are the LST products used by Copernicus Global Land Service.

Supplementary Table 2: Description of deforestation experiments in CESM 1.2.2

Name	Component set	Resolution	Name	Land cover
Low-res offline deforestation	I_2000 (CLM4 with the NCEPCRU forcings)	f19_g16 (1.9°×2.5°)	Pre-deforestation	Pre-industrial condition in 1850
			Post-deforestation	Complete deforestation
Low-res coupled deforestation	F_2000 (CLM4 with CAM4)	f19_g16 (1.9°×2.5°)	Pre-deforestation	Pre-industrial condition in 1850
			Post-deforestation	Complete deforestation
Low-res coupled subgrid deforestation	F_2000 (CLM4 with CAM4)	f19_g16 (1.9°×2.5°)	Pre-deforestation	Pre-industrial condition in 1850
			Post-deforestation	Only 0.01% tree PFTs left after deforestation
High-res coupled deforestation	F_2000 (CLM4 with CAM4)	F05_g16 (0.47°×0.63°)	Pre-deforestation	Pre-industrial condition in 1850
			Post-deforestation	Complete deforestation

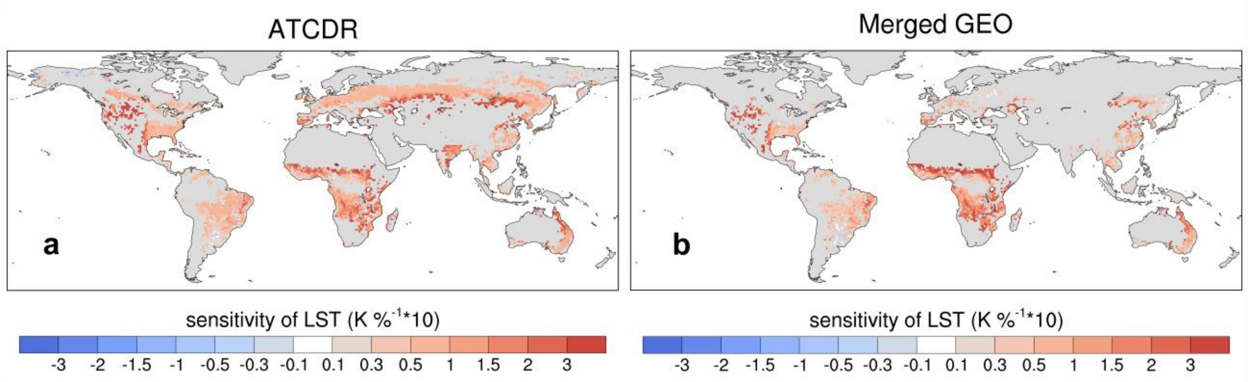

Supplementary Figure 1: Observed sensitivity of summer daytime land surface temperature deforestation. **a** the ATSR Climate Data Record (CDR) product derived from Along Track Scanning Radiometers 2 (ATSR-2) and Advanced Along Track Scanning Radiometer (AATSR), and **b** the GEO land surface temperature (LST) merged product retrieved from three geostationary satellites (GOES over America, SEVIRI over Europe and Africa, and MTSAT/HMWR over eastern Asia and Australia). The sensitivity is calculated based on different satellite products within a 1°×1° analysis window. The sensitivity is shown only where it is significant at a False Discovery Rate (FDR) adjusted p values of 0.05 and the range of deforestation (maximum minus minimum deforestation within the window) is greater than 10%.

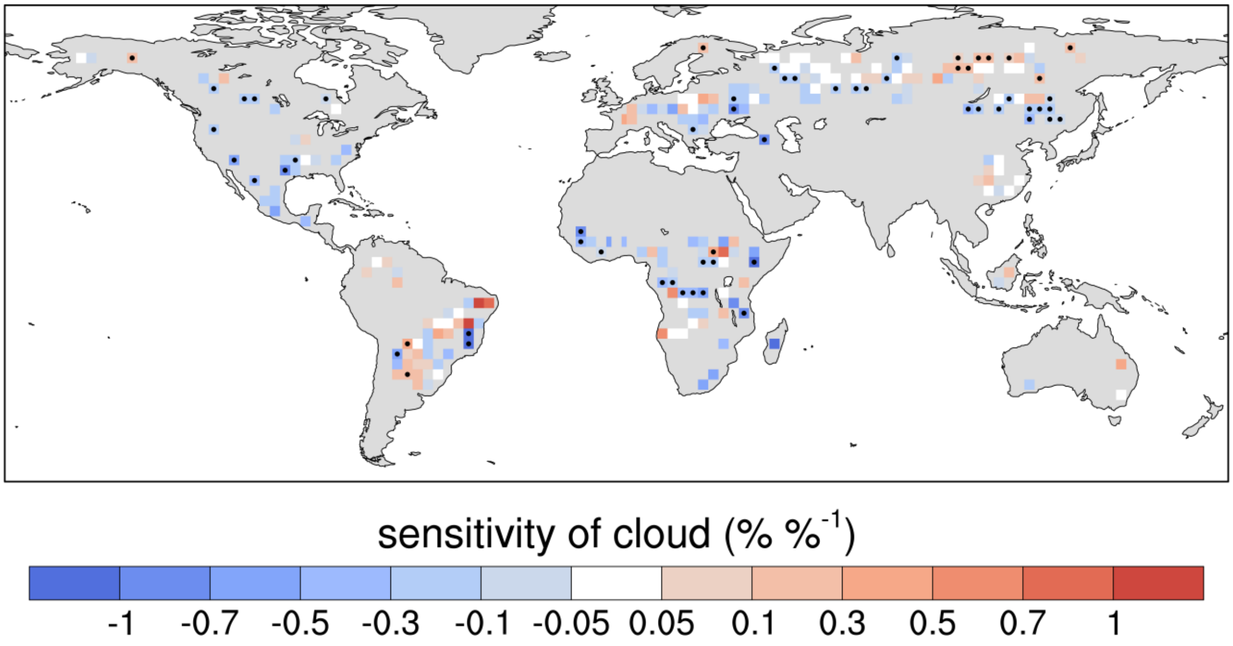

Supplementary Figure 2: Observed sensitivity of cloud cover to deforestation. The sensitivity is calculated based on MODIS monthly cloud fraction product (MOD08_M3) within a 3°×3° analysis window. The black dot signs indicate the sensitivity is significant at a False Discovery Rate (FDR) adjusted p values of 0.05.

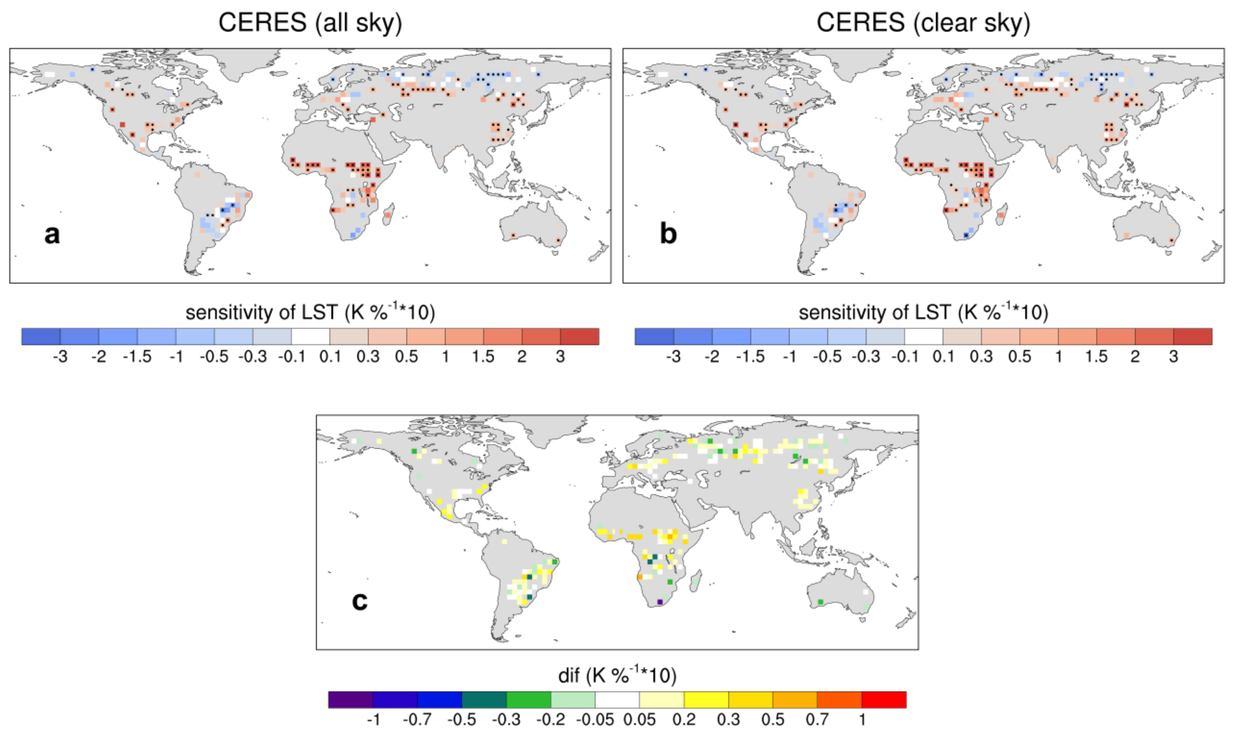

Supplementary Figure 3: Observed sensitivity of summer land surface temperature to deforestation. The sensitivity is calculated based on the Clouds and the Earth's Radiant Energy System (CERES) upward longwave radiations under **a** all-sky (the same as Fig. 5e) and **b** clear-sky conditions; **c** is their difference (all-sky minus clear-sky). The black dot signs indicate the sensitivity is significant at a False Discovery Rate (FDR) adjusted p values of 0.05.

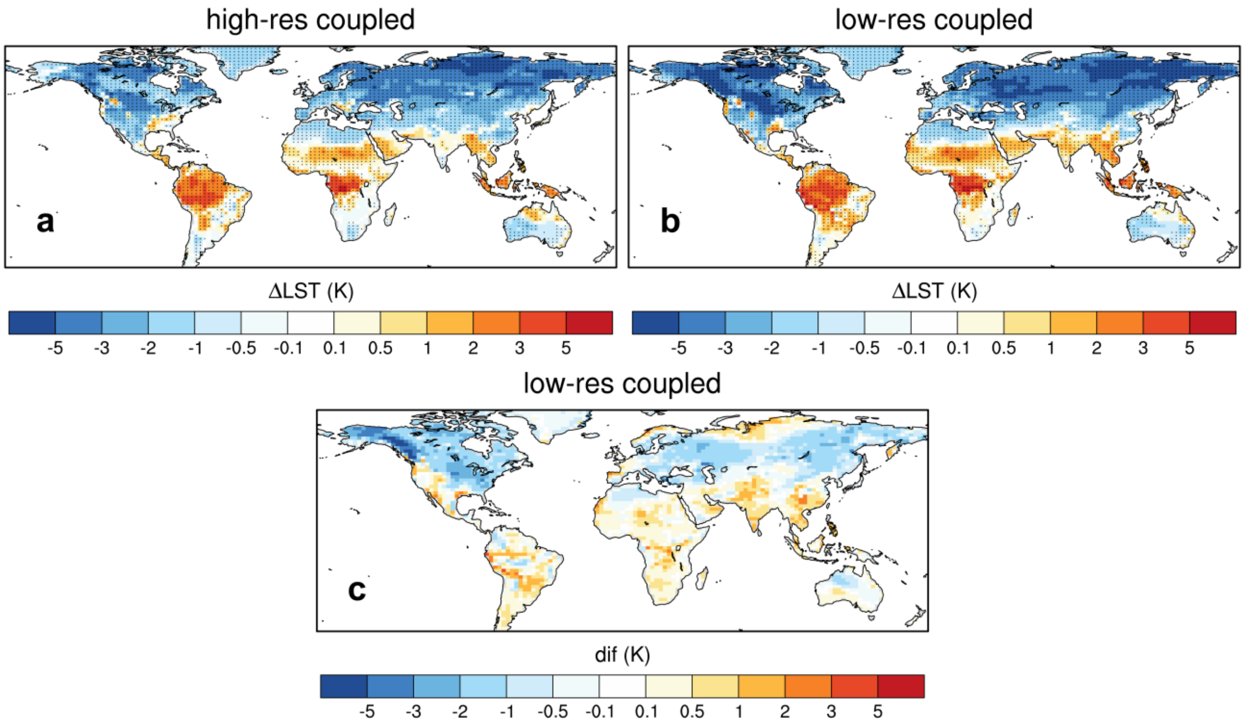

Supplementary Figure 4: Simulated changes in summer daily maximum land surface temperature. The temperature changes (ΔLST) are calculated based on **a** high-res and **b** low-res coupled deforestation experiments; **c** is their difference (low-res minus high-res). The high-res results are aggregated to the same resolution as the low-res results for comparison. Stippling indicates the temperature difference is significant at a False Discovery Rate (FDR) adjusted p values of 0.05.

Supplementary References:

1. Wan, Z., Hook, S., Hulley, G. MYD11C2 MODIS/Aqua land surface temperature/emissivity 8-Day L3 global 0.05Deg CMG V006. NASA EOSDIS Land Processes DAAC. (2015).
2. Didan, K., Munoz, A.B., Solano, R. and Huete, A. MODIS vegetation index user's guide (MOD13 series), version 3.0.0 (collection 6). Tucson,AZ: Vegetation Index and Phenology Lab, The University of Arizona,32 pp. https://vip.arizona.edu/documents/MODIS/MODIS_VI_UsersGuide_June_2015_C6.pdf (2015).
3. Schaaf, C. and Wang, Z. (2015) MCD43A1 MODIS/Terra+AquaBRDF/albedo model parameters daily L3 global-500m V006. NASAEOSDIS Land Processes DAAC. https://doi.org/10.5067/MODIS/MCD43A1.006 (2015).
4. Mu, Q., Zhao, M. & Running, S. W. Improvements to a MODIS global terrestrial evapotranspiration algorithm. *Remote Sensing of Environment* **115**, 1781-1800 (2011).
5. Du, J., J. S. Kimball, L. A. Jones, Y. Kim, J. Glassy, and J. D. Watts. A global satellite environmental data record derived from AMSR-E and AMSR2 microwave earth observations, *Earth System Science Data*. **9**, 791-808 (2017).
6. Gelaro, R., et al. The Modern-Era Retrospective Analysis for Research and Applications, version 2 (MERRA-2). *J. Climate*, **30**, 5419–5454 (2017).
7. Coccia, G., Siemann, A.L., Pan, M., Wood, E.F. Creating consistent datasets by combining remotely-sensed data and land surface model estimates through Bayesian uncertainty post-processing: The case of Land Surface Temperature from HIRS. *Remote Sensing of Environment*. **170**, 290-305 (2015).
8. Dee, D., et al. The ERA-Interim reanalysis: Configuration and performance of the data assimilation system. *Quarterly Journal of the royal meteorological society*. **137**, 553-597 (2011).
9. Wielicki, B. A. *et al*. Clouds and the Earth's Radiant Energy System (CERES): An earth observing system experiment. *Bull. Amer. Meteor. Soc.* **77**, 853-868 (1996).
